# SimulatorOrchestrator: A 6G-Ready Simulator for the Cell-Free/Osmotic Infrastructure†

**DOI:** 10.3390/s25051591

**Published:** 2025-03-05

**Authors:** Rohin Gillgallon, Reham Almutairi, Giacomo Bergami, Graham Morgan

**Affiliations:** 1 School of Computing, Faculty of Science, Agriculture and Engineering, Newcastle University, Newcastle upon Tyne NE4 5TG, UK or reham.mutlaq@uhb.edu.sa (R.A.); giacomo.bergami@newcastle.ac.uk (G.B.); graham.morgan@newcastle.ac.uk (G.M.); 2 College of Computer Science and Engineering, University of Hafr Al Batin, Hafr Al Batin 31991, Saudi Arabia

**Keywords:** IoT, healthcare transportation, 6G architecture, traffic simulators, patient monitoring

## Abstract

To the best of our knowledge, we offer the first IoT-Osmotic simulator supporting 6G and Cloud infrastructures, leveraging the similarities in Software-Defined Wide Area Network (SD-WAN) architectures when used in Osmotic architectures and User-Centric Cell-Free mMIMO (massive multiple-input multiple-output) architectures. Our simulator acts as a simulator orchestrator, supporting the interaction with a patient digital twin generating patient healthcare data (vital signs and emergency alerts) and a VANET simulator (SUMO), both leading to IoT data streams towards the cloud through pre-initiated MQTT protocols. This contextualises our approach within the healthcare domain while showcasing the possibility of orchestrating different simulators at the same time. The combined provision of these two aspects, joined with the addition of a ring network connecting all the first-mile edge nodes (i.e., access points), enables the definition of new packet routing algorithms, streamlining previous solutions from SD-WAN architectures, thus showing the benefit of 6G architectures in achieving better network load balancing, as well as showcasing the limitations of previous approaches.The simulated 6G architecture, combined with the optimal routing algorithm and MEL (Microelements software components) allocation policy, was able to reduce the time required to route all communications from IoT devices to the cloud by upto 50.4% compared to analogous routing algorithms used within 5G architectures.

## 1. Introduction

The healthcare sector exploits Internet of Things (IoT) technologies to improve both patient care and health outcomes by leveraging the basics of smart city infrastructures [1,2]: equipping ambulances with IoT sensors to transmit patients’ condition from ambulances to hospitals allows medical staff at the hospitals to determine and prepare any necessary patient treatment in advance [3,4,5]. Ramesha et al. [6] extend this by connecting ambulances to traffic control units for uninterruptedly routing the ambulances through synchronised traffic lights, which might be turned green alongside the route to the hospital, thereby reducing the time needed to get patients to the hospitals. Given the time-sensitive nature of ambulances rushing patients to hospitals, it is equally essential that the patient data are transmitted from the IoT-equipped ambulances to the hospitals as quickly as possible to give as much time as possible to the medical staff at the hospitals to prepare for incoming patients adequately. Due to the mobile nature of ambulances, the straight transmission of data from the ambulances to the hospitals is likely to be unstable due to unpredictable environmental obstacles, high vehicle speeds, and traffic congestion. These issues lead to highly dynamic network topologies, requiring roadside units (RSUs) as edge nodes to ensure a constant, stable network connection for the ambulances. RSUs can then collect patient data from nearby ambulances and forward them to the relevant hospitals for processing. In e-health applications, particularly during emergency medical transportation, the Quality of Service (QoS) requirements are stringent, with a necessity for low-latency and high-reliability communication channels. This ensures that patient data transmitted from IoT-enabled ambulances to hospitals are received without delay or loss, enabling timely medical interventions [7]. To ensure the effectiveness of IoT systems in real-world settings, researchers have stressed the importance of accurately modelling and simulating such systems [8]. As more patient data will be streamed and collected within smart cities, the network infrastructure must handle the data transfer and processing of the transmitted information in real time, where both mobile and static IoT devices coexist. The patient data will include real-time data collected from patients’ wearable devices [9] while at home, creating an extremely interconnected scenario which will severely increase the amount of Internet traffic within the Osmotic infrastructure. In the context of such healthcare transportation research, advanced simulation platforms are critical to evaluating and improving patient transportation strategies, including testing the necessary network infrastructure. Despite the importance of trace data for network simulators, there is limited public availability [10,11,12], which has led to the generation of traces either from real-world traffic data or, in some cases, synthetic traces generated using simulators. This research extends SimulatorBridger [13] into SimulatorOrchestrator (https://github.com/LogDS/SimulatorOrchestrator/releases/tag/v1.0, accessed on the 1 March 2025) to allow for the direct ingestion of real-time IoT communication events coming from another simulator external to the VANET, while also considering possible minimal extensions of the simulator enabling a 6G infrastructure via cell-free networks. As a use case of the former, we consider patients’ digital twins generating traffic to update clinicians via the cloud in real time on the health conditions of the patient recovering at home. This is an important step, as it showcases the possibility of extending this simulator to a general-purpose orchestrator of different simulators, contextually generating events of interest.

As with [1], the UK’s new NHS (National Health Service) electric vehicle initiative to help reduce its carbon footprint [14] was a driving force behind choosing the healthcare sector for the simulated use case of SimulatorOrchestrator. As a result of these advancements, our previous work related to SimulatorBridger [1] was extended to handle patient health information transmitted by IoT devices during the transport process and accurately simulate scenarios in which electric ambulances (EAs) are used to transport patients whilst ensuring the timely and secure transmission of health data. This motivates our extension of the former to further support patient IoT data beyond vehicular patterns. In the present paper, we extend the former solution to include new communication events that might be while also including data that can be streamed in real time by home patient care requiring ad hoc monitoring. Adding an external patient simulator required us to extend the simulator further to address the possibility of making multiple simulators interact and be orchestrated to simulate one scenario of interest. This required a major architectural restructuring of the initial work, thus leading to a novel simulator named SimulatorOrchestrator (See RQ №2.).

**Figure 1:**
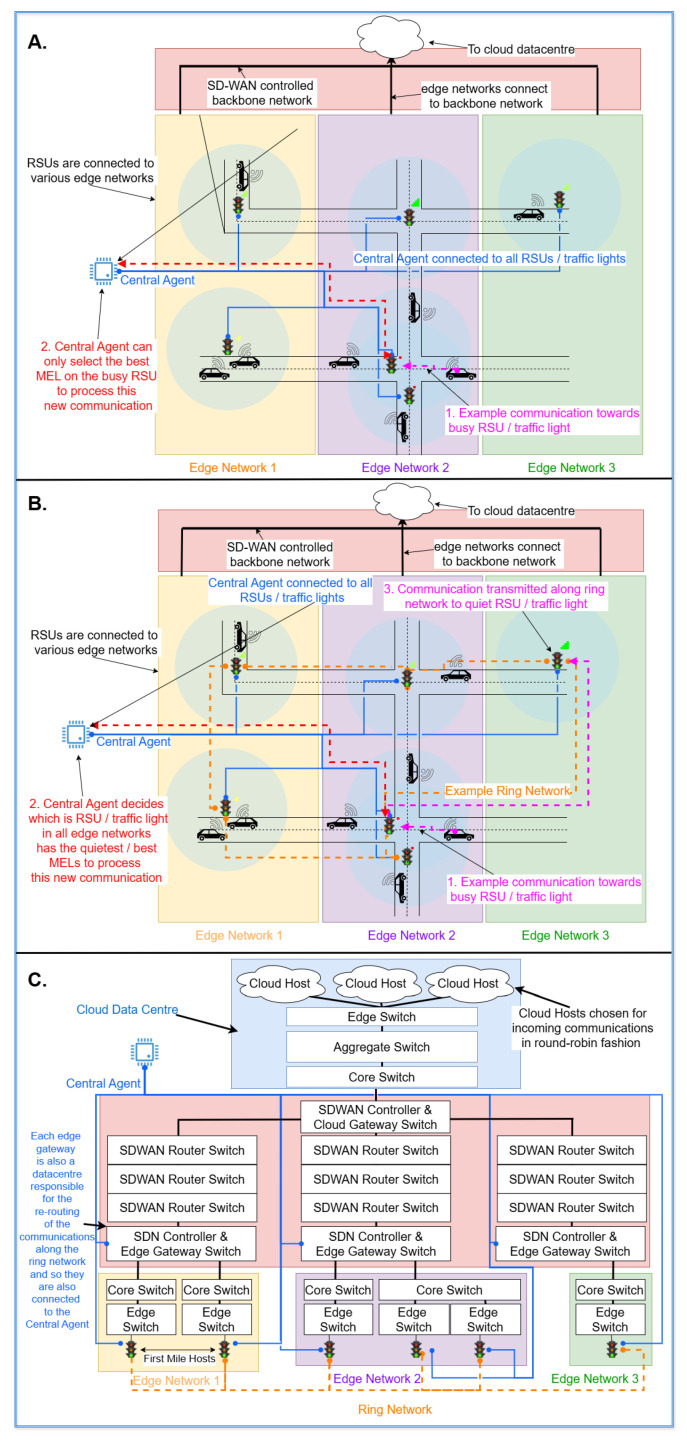
Showcasing the main differences in the routing algorithms between the ones from the Osmotic architecture [1] (**A**) and the others from our proposed Osmotic/cell-free architecture ((**B**), Section 4). (**C**) shows the main architectural overview of all the sub-networks simulated within our meta-simulator (Section 3.3.3). The colours of the lines and the text are coordinated, the colour of the text indicates which aspect of the plot they are explaining - and the numbered texts are to be read in ascending order.

From the communication architecture perspective of a 5G network supporting SD-WAN Osmotic infrastructure, this paper addresses the main limitation of our previous work [1]. The choice of MicroELements software component [15] (MEL) allocation policy originally had no impact on network behaviour. If a communication was initiated from a device near a busy edge node with bottlenecked MELs, it had to be processed by one of those bottlenecked MELs. If all the MELs were bottlenecked, no impact should be expected. By leveraging the 6G architecture and by assuming that all the first-mile edge nodes (APs being RSUs) are connected in a metropolitan area network (MAN) token ring network, we aim to load balance the transmission of packages better; we can avoid bottlenecking by re-forwarding the packet to a “quieter” edge subnetwork, i.e., a network having less traffic allocation (Figure 1). Our paper shows the effectiveness of exploiting the Million Instruction Per Second (MIPS) allocated to each MEL component supporting the packet transmission (Section 3.3.3). The paper addresses the following research questions:

**RQ №1.**  It is possible to minimally extend an Osmotic Simulator also to support 6G cell-free architecture, comparing ‘A’ with ‘B’ on Figure 1: After observing that *(i)* both can operate on software-defined networks (SDN) [16,17], *(ii)* there exists a parallelism between edge nodes associated with computing abilities (Section 2.2) [18] and the user-centric cell-free massive multiple-input multiple-output (UC CF-mMIMO) access points (APs) (Section 2.1), *(iii)* the parallelism between Osmotic Central Agents [13,18] and cell-free Central Processing Units [19,20,21], and *(iv)* given that SimulatorOrchestrator supports all of the aforementioned Osmotic architecture features via SimulatorBridger [13], then our previous simulator SimulatorBridger can be minimally extended also to simulate cell-free architectures (Section 3.2). By assuming almost simultaneous communication across the edge nodes belonging to the same MAN through a Gigabit Token Ring (Section 3.3.3), it is possible to establish connections across different area networks, thus breaking the rigid cell and edge network structure.
**RQ №2.**  It is possible to orchestrate a network simulator with another one providing additional packet transmission events to the former: By extending the interface of our previous simulator, SimulatorBridger, to run only within subsequent time intervals (Section 3), and by allowing the possibility of pause for then resuming the simulation, it is possible to concurrently run it alongside other simulators, generating potential new packet transmission events (Section 3.2). In this paper, we take into consideration patient digital twins transmitting healthcare data to the cloud (Section 3.3.2) running on a pre-initiated MQTT protocol (Section 2.3), and separated from vehicular ad hoc network (VANET) or Connection Counting [1] inputs for vehicular traffic (Section 3.3.1).
**RQ №3.**  The combined provision of SD-WAN and cell-free architectures enables efficient packet routing algorithms ensuring QoS: Given the fulfilment of RQ №1., we propose a new MEL allocation policy crossing the boundaries of a single-edge network (Section 4.3) which, when combined with our previously introduced MCFR algorithm (Section 4, [22]), effectively reduces the number of active communications while increasing the packet rate (Section 5). This solution outperforms routing algorithms working on a 5G architecture not working on a cell-free assumption. This is achieved by centralising the execution of the routing algorithm at the Central Agent/central processing unit (CPU) level rather than at the single-edge gateway, as in [17], thus allowing the re-route of each packet to be sent to the cloud via a specific “quiet” (e.g., non-busy) network. On Figure 1, ‘C’ shows a simplified model of our simulated network architecture, as we route communications from the IoT devices to a cloud data centre via edge and backbone networks.

We investigate this by generating realistic traffic patterns expressed as mobility traces (MTs) through SUMO. We choose to consider those occurring in Bologna (https://github.com/DLR-TS/sumo-scenarios/tree/main/bologna/acosta, accessed on 1 March 2025), a renowned healthcare smart city, which was extended by placing three patients, one with a higher risk factor than the previous one, at 15 of the 16 edge nodes in the smart environment. At the final edge node, we placed five patients for each risk level to model a hospital with patients at different recovery levels.

## 2. Related Works

### 2.1. 6G Architecture

Recent work [23] highlights the potential of 6G in medical contexts, proposing an architecture where modular application functions (MAFs) dynamically interact with network resources, adapting their requirements based on current network conditions. This capability is especially relevant for healthcare, as it enables resource allocation adjustments in real time to prioritise critical applications such as remote patient monitoring and emergency response.

One of the primary potential technologies for 6G is cell-free massive multi-input multi-output architecture (CF-mMIMO) [24]. The key idea behind CF-mMIMO is the distribution of access points (APs) throughout an environment to create a sizeable homogenous coverage area. This technology aims to combat issues with cell-based architectures, including inter-cell interference and signal degradation as a device gets further away from the cell centres [25,26]. An initial assumption within CF-MIMO systems was that all devices should be able to access all APs to overcome user performance and location-based issues. However, this results in problems scaling as the number of connecting devices grows, such as high computational complexity and high power consumption. To tackle this scalability issue, the user-centric CF-mMIMO (UC CF-mMIMO) has been proposed, where devices now need only connect with a smaller number of nearby APs. UC CF-mMIMO tackles the scaling issues while maintaining consistent coverage for all devices within the networked area [25].

Within cell-free-based environments, the APs are connected to a central processing unit (CPU) that receives the data via the APs and processes all the available data [25,26,27]. To improve this architecture, mobile edge computing (MEC) technologies can be combined with the APs within a UC CF-mMIMO by equipping the APs with MEC servers, as was the case with [28,29]. Within the MEC UC CF-mMIMO, devices can connect to a nearby AP to transmit the data; the data can then be processed/preprocessed before being transmitted towards a central server/processing unit. The incorporation of MEC servers with AP networks obtains the combined benefits of cell-free-based technologies tackling problems with signal degradation and inter-cell interference with the benefits of MEC computing, which help tackle the ‘critical latency requirements of computationally intensive tasks’ introduced with 5G technologies [28]. Within these MEC CF-mMIMO, the APs can remain connected to the CPUs via reliable front-haul or back-haul links, either wired or wireless, depending on what best suits both the network topology and environmental topology [21,28]. Within UC CF-mMIMO, a cluster of APs can be assigned to a single MEC server, and therefore, it is also possible to have multiple MEC servers, each with a cluster of APs. It is then important for the MEC servers to be able to manage their APs correctly. This can be achieved through the use of distributed software-defined networks (SDNs) [16], as they can control the utilisation of both the MEC server resources, as well as its associated APs, through the use of software; for example, routing the incoming data towards the APs through the network towards its destination, e.g., a cloud data centre.

Within UC CF-mMIMO, it can be beneficial for the efficiency of the network for all APs to be connected to a central processing unit that has perfect knowledge of the channel state information (CSI) within the network to aid with the network and planning [19], thus coordinating the communications from the IoT devices connected to the APs [20]. This is similar in concept to the Central Agent in Osmotic computing [18], which makes decisions on the operations performed by the other agents within the network.

By leveraging the flexibility of a cell-free network architecture in 6G, which adopts a user-centric design coupled with MEC-equipped APs/RSUs as edge nodes, we anticipate our simulator can provide seamless and high-quality connections for urban healthcare settings where smart devices rely on stable and continuous connectivity [30]. This paper aims to place the simulator at the forefront of healthcare IoT research, incorporating advanced communication frameworks that support highly responsive, data-intensive healthcare systems in smart city environments by challenging minor possible extensions and assumptions at their boundaries while determining whether those will suffice to achieve the 6G requirements.

### 2.2. IoT and Osmotic Simulators

IoT and Osmotic (edge/cloud) simulators are currently used to simulate network traffic conditions under different network and resource allocation policies for streamlining real-time communications [31]. As a compromise from modelling and simulating complex communication patterns over many devices, simulators such as [18,32] do not fully simulate all seven Open Systems Interconnection (OSI) layers while focusing on the most relevant ones. This constraint is born of necessity due to the vast range of requirements and protocols found across disparate IoT devices, adding additional complexity to any simulation aiming to comprehensively simulate any, let alone all, of the OSI layers. Concerning the IoT and Osmotic simulator presented in the current paper, the Presentation and Session layers simulation took between 40 min and 2 h and 30 min real time to simulate just 300 s (5 min). Given this disparity, attempting the simulation of all OSI layers would have been impractical, even if it would have led to a more realistic network and a more complete simulation. However, aside from simply losing increased realism, not simulating the full OSI stack comes at the expense of lacking any information which may be necessary for simulating cybersecurity-related VANET scenarios, such as battery-draining attacks [33]. Furthermore, each OSI layer has its vulnerabilities, meaning for the current state-of-the-art to accurately determine how susceptible a given network topology is to attacks, all OSI layers would need to be simulated [34]. Such scenarios are outside the scope of this work, and so focusing on the Session and Presentation layers was deemed to be sufficient for this work, as our major interest is to determine how network infrastructure and specific network routing policies might drastically affect the communication delays within smart city scenarios runnon on Osmotic and SDN assumptions. Last, to the best of our knowledge, no IoT and Osmotic simulator was currently extended to also support 6G architectures.

### 2.3. MQTT Protocol

The MQTT publish–subscribe model is identified as a suitable real-time monitoring solution, particularly effective for mobile healthcare applications like ambulances and dynamically changing vehicular networks. This lightweight protocol ensures efficient data transfer with minimal delay, critical for transmitting patient data and vehicular traffic information in real time [35]. MQTT helps streamline real-time monitoring systems by reducing bandwidth usage and improving response times, prioritising only necessary data for immediate processing. Research on cooperative driving communications demonstrates the importance of timely data exchange for coordinated and ethical vehicle behaviours, highlighting the need for low-latency, real-time communication in similarly dynamic environments [36]. Similarly, recent studies on scalable network solutions for large UAV swarms emphasise the importance of efficient data management across adaptive networks, underscoring the value of robust algorithms to handle data flow in large-scale, fast-changing systems [37]. Given this, the simulator freely assumes that each patient and vehicle within the simulation already went through the preliminary phase of the protocol, thus only simulating the par where each IoT device (both patient and vehicle) publishes the content to the MQTT broker being distributed across the network across all the cloud virtual machines.

## 3. SimulatorOrchestrator

### 3.1. Simulator Overview

The foundation of SimulatorOrchestrator is CloudSim 3.0.3 [38], a Java-based, discrete-event simulation toolkit used in the development and evaluation of different resource-management strategies in cloud computing scenarios whilst allowing for the simulation of data centres and virtual machines (VMs) [17]. CloudSim has become the de facto standard when simulating such scenarios, with the initial paper being cited over 6400 times [39]. Given that CloudSim’s focus is on the cloud, other works have built upon CloudSim to simulate a wider range of scenarios. One such example is IoTSim [40], which added the functionality to simulate multiple simultaneous IoT applications to help the processing of big data.

An extension more relevant to our work is IoTSim-SDWAN [17], which added the capabilities to model “*multiple SDN-enabled data centres running within SD-WAN environments*”. The work of [17] developed a simulation tool allowing for the simulation of “*SD-WAN ecosystems and SDN-enabled multi-cloud environments*”. IoTSim-Edge [41] recontextualised the functionality of CloudSim to model communications between IoT devices and hosts, modelling the Cloudlets from CloudSim as Edgelets and the VMs as MELs. IoTSim-Osmosis [42] then combined the features and functionality from both IoTSim-SDWAN [17] and IoTSim-Edge [41] to create an “*SDN-based osmotic computing toolkit*” modelling communications from IoT devices toward the edge-cloud continuum. This was then developed further by [18] to create IoTSimOsmosis-RES, adding the simulation of renewable energy sources for powering the IoT devices. The work of [18] also added Osmotic agents to the IoT devices as well as the edge and the cloud data centres, which allows for the implementation of a MAPE (Monitor, Analyse, Plan, and Execute) loop, which allows for better control and algorithmic management of these aspects of the network. In particular, the present work leverages this controlling mechanism to dynamically re-route the communications happening within the network to improve the Quality of Service (QoS).

SimulatorBridger [13] bridged for the first time IoTSimOsmosis-RES (https://github.com/tszydlo/IoTSim-Osmosis-RES, accessed on 1 March 2025) using CloudSim 3.0.3 with an external mobility simulator, SUMO 1.21.0, allowing for the simulation of IoT devices communicating dynamically in urban areas. We simulated vehicles communicating with roadside units as first-mile edge nodes. These edge nodes then route communications to a cloud data centre through the SDN network. Most recently, in addition to the connection counting from [1], within [22], we added the simulation of the 3G, 4G, and 5G cellular networks to SimulatorBridger.

### 3.2. SimulatorOrchestrator for 6G Cell-Free Architectures

**Figure 2:**
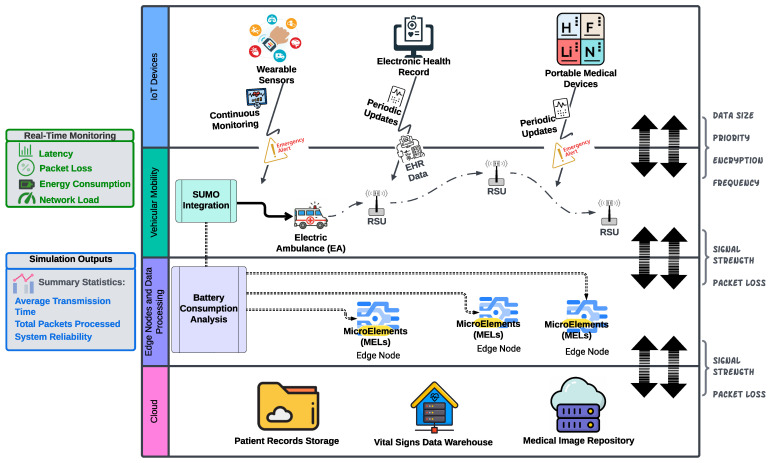
Agent components supported in SimulatorOrchestrator: the cloud and edge components are simulated through SimulatorBridger (Section 3.3.3), Vehicular Mobility Patterns are obtained via SUMO (Section 3.3.1), while other IoT devices except the ones associated with the vehicular traffic are generated from the patient digital twin (Section 3.3.2).

**Figure 3:**
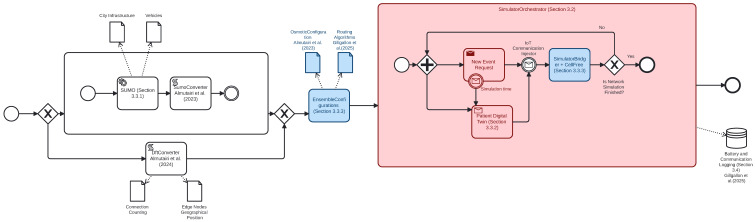
BPMN Diagram illustrating the proposed architecture for SimulatorOrchestrator: parts in blue remark extensions from previous work, and ones in red reveal novel additions to the architecture [1,13,22].

This paper postulates the possibility of achieving 6G architecture by extending an Osmotic architecture to encompass cell-free infrastructure simulation while improving the connection across all the nodes belonging to the edge communication layer. To this end, we also show the need to extend the routing policies further to acknowledge the proposed network infrastructure. To demonstrate this, we extend our Osmotic architecture from SimulatorOrchestrator, already encompassing the combination of a VANET simulator for vehicular traffic with an Osmotic architecture simulator, by also considering patient digital twins generating additional packets with different levels of urgency, vehicular mobility, edge computing, and Osmotic computing in a smart city environment providing a cell-free infrastructure. The generator is designed to handle situations in which real-time healthcare data are collected, transmitted, and processed on the cloud. At the same time, patients are transported in vehicles equipped with IoT-enabled medical devices, such as electric ambulances (EAs). While Figure 2 describes the simulator from the perspective of the agents being simulated within the software infrastructure, Figure 3 describes the simulator as an orchestrator across different simulators.

SimulatorOrchestrator procedurally generates the network infrastructure by considering either the RSU distribution within a city environment [13] or by precisely providing the exact location of their position [1]. Each edge subnetwork is created from a cluster of connected components that can easily share the traffic information and constitute a single communication island utterly detached from the othercells (EnsembleConfigurations). While the first configuration is the preferred choice when we possess more precise traffic vehicular information, the latter is used when we are interested in approximate IoT communications through connection counting. We consider both solutions. In particular, when running SUMO to obtain precise vehicular information, we generate vehicular traces per vehicle in the form of location-time-step pairs (LTSPs). These data were then stored in a SQL table [22], which will be then used by the main SimulatorBridger network communication simulator; LTSPs lead to the scheduling of “wake-up” simulator time events, i.e., times at which SQL data are to be injected into SimulatorBridger allowing for the positions of the vehicles to be checked and initiating any possible communication from a vehicle if near to a first-mile edge node (i.e., RSU as AP), thus reaching the nearest one [13]. This approach is only suitable for either simple situations where the results can be computed at runtime or deterministic simulators, with no dynamic behaviours, under the assumption that the cars will not change their routing algorithm depending on the possible events generated by the main networking simulator (SimulatorBridger).

However, one of the goals of SimulatorOrchestrator is the ability to combine and connect multiple simulators with the IoT simulator, which would potentially need to adapt their behaviour depending on the current network status. Among those, we can also consider non-deterministic simulators relying on complex decisions that cannot simply be computed at runtime, as well as others requiring access to the current state of a different simulator to be used in their decision-making. In such cases, it is clear that simply running each simulator before running the main Osmotic simulator and simply injecting the traces of the vehicles on the Osmotic simulator would not result in a realistic setup. This then requires that any simulator require dynamic updates to be run simultaneously with the main networking architecture and allow any newly generated communication initiation step to be injected in SimulatorBridger. In our case, a patient’s health does not affect vehicle traffic or vice versa, so we do not need to account for how these two aspects interact, meaning we can still use the traffic simulator’s batched output. The former also makes a sensible assumption in the context of our simulator, as we focus more on testing the possibility of defining efficient routing algorithms for the Osmotic/cell-free 6G architecture for streamlining communications rather than faithfully representing patient data, as this is not our primary target of analysis. On the other hand, the network traffic from each scenario interacts and impacts the whole network, so the data from the traffic simulator and the patient health data must be injected into the IoT simulator.

The main impact of extending the previous simulator architecture so as to encompass an orchestration of multiple simulators running at the same time is highlighted in red in the BPMN diagram from Figure 3: after an initialisation step, which encompasses the generation of the networking architecture from either the connection counting strategy or from a VANET simulator such as SUMO, the data resulting from this are sufficient to run the previous SimulatorBridger entirely by considering vehicular updated positions via LTSP. The network simulation then finishes when there are no more networking events to be handled. When this happens, a shutdown process is started, after which we collect further network statistics [18]. To allow the interaction with other simulators, SimulatorOrchestrator will require the definition of a simulation time granularity interval δ, after which the Osmotic simulator being SimulatorBridger is temporarily suspended. This results in an event dispatch request towards the other concurrent simulators (e.g., the patient digital twins), which might generate new communication events to be injected while running SimulatorBridger occurring within the time frame of the forthcoming time intervals. When the other concurrent simulators terminate the generation of new potential communication events, the computation of SimulatorBridger is then resumed, thus continuing to handle other requests. At the time of writing, the last communication time will still be determined by the LTSP with the greatest time value, after which no further requests to the other concurrent simulators are dispatched while waiting to handle the network shutdown event.

### 3.3. Simulation Input

We now discuss the different kinds of simulation inputs currently supported by our simulator, overall orchestrating a vehicular (Section 3.3.1) and a patient digital twin (Section 3.3.2) into the main backbone constituted by a Cell-Free Osmotic Simulator (Section 3.3.3), which is ready for simulating futuristic 6G Architectures before deploying them in the real world.

#### 3.3.1. Vehicular Mobility Patterns (SUMO, Connection Counting)

When patients are transported by smart vehicles, such as electric ambulances (EAs), the data are continuously transmitted to nearby RSUs/APs/first-mile edge nodes positioned along their route. By integrating the proposed health data generator with the traffic generator, SUMO [43], the system can accurately simulate the movement of EAs, maintaining the dynamic nature of vehicular mobility. SUMO provides realistic mobility traces (MTs), which are critical for linking the dynamic location of IoT-enabled devices on ambulances to the generated healthcare data streams.

For example, the VANET generator simulates scenarios where an ambulance transmits patient health information to a hospital while navigating through urban traffic, using SUMO’s realistic simulation of vehicular movement. However, while the simulator can directly control and track the routes of smart electric ambulances (EAs), as in SUMO, interactions with civilian vehicles introduce additional complexities in traffic flow that cannot be fully predicted or controlled. Therefore, to account for the unpredictable nature of civilian traffic, it is essential to incorporate vehicular connection counting, as discussed in our previous work [1]. This approach enables the integration of both controlled traffic from EAs and uncontrolled civilian traffic, creating a more comprehensive simulation that accurately reflects the complexities of urban mobility and its impact on healthcare data transmission.

#### 3.3.2. Dynamic/Concurrent Simulators (Patient Digital Twin)

The key difference between a digital twin and a simulator is the following: while the latter mainly replicates what might happen to a product, the digital twin replicates what is happening to the entity of interest while considering the world surrounding them as a precious context, where the agents’ statuses can also be probed through sensors (https://www.twi-global.com/technical-knowledge/faqs/simulation-vs-digital-twin, accessed on 1 March 2025). This makes digital twins the ideal solution to simulate patients in healthcare scenarios. In our scenario, we are still considering a simplistic patient digital twin generating health information for each patient of interest, such as vital signs in patient records; emergency alerts are handled by increasing the rate at which vital sign information is transferred through the network to the cloud.

We consider MedStream Analytics (https://github.com/IshaanAdarsh/MedStream-Analytics, accessed on 1 March 2025) as our preliminary basis for our patient digital twin providing randomly generated heart rate (*h*, in beats per minute (bpm)), blood pressure (*p*, in mm Hg), and body temperature (*t*, in °C). Vital sign values are randomly generated using a Gaussian distribution, which considers mean and standard deviation values for healthy and ill patients according to the current medical literature [44,45,46]. Given $1_{P}$ the indicator function [47] returning 1 if the condition stated in *P* holds and 0 otherwise, the monitoring model considers each of the vital signs of interest (*h*, *t*, and *b*) to provide a health risk score τ as follows:

$$\left\{\begin{array}{cc} hr=1_{h\leq 50}+1_{h\leq 40}+1_{h\geq 91}+1_{h\geq 110}+1_{h\geq 131} & \\ tr=1_{t\leq 36}+2\cdot 1_{h\leq 35}+1_{h\geq 38.1}+1_{h\geq 39.1} & \\ br=1_{p\leq 110}+1_{p\leq 100}+1_{p\leq 90}+3\cdot 1_{p\geq 220} & \\ \tau =hr+tr+br \end{array}\right.$$

Healthy patients with τ≤3 transmit their data to the Central Agent every 10 s; however, once the patient’s total risk value exceeds a given threshold, they are considered at risk, transmitting their data every second. This increased update rate aims to provide health professionals with the most up-to-date information on the vital signals from at-risk patients. Our simulation system’s core, as shown in Figure 2, considers patients as IoT devices continuously monitoring and collecting health-related data. Quality of service (QoS), including low latency and high reliability, is essential for timely and accurate medical responses and better patient data monitoring.

#### 3.3.3. Main Cell-Free/Osmotic Architecture Simulator (SimulatorBridger)

Within SimulatorOrchestrator, we are modelling a Software-Defined Wide-Area Network (SD-WAN) architecture with multi-data-centres [17]. In a smart city scenario, first-mile edge nodes, represented as AP over RSUs, receive data from the IoT devices and forward their packets to the cloud, where the packets will be processed inside a virtual machine. For each edge network, we have a main data centre and SD-WAN controller acting as a network gateway, thus connecting it to the backbone network. As such, the SD-WAN controller component of such a gateway is responsible for managing and allocating a communication channel to enable effective packet transmission. Thus, the SD-WAN is also responsible for running different routing algorithms [22] and re-pathing the packet communication channel if required [17]. Edge networks all have the same resources but different numbers of hosts. Each edge network has the same maximum number of hosts; their first-mile edge nodes are connected to the main cloud data centre through switches that can be differentiated into aggregate switches, which are connected to core switches, which finally connects to a gateway on the backbone network. As a result, each edge network is a Tree Network [48]. As the backbone network connects each edge network to the main cloud network, such gateways ultimately enable each IoT packet to reach the cloud. Upon reaching the cloud, data are processed and stored more efficiently, enabling a deeper analysis and longer-term storage. Furthermore, the cloud infrastructure provides a feedback loop through which insights and processed data can be sent back to first-mile edge nodes or IoT devices in real time, enabling real-time decision-making and adjustments to patient care as necessary. Additionally, given SimulatorOrchestrator employs Osmotic computing, the IoT devices do not directly interact with the first-mile edge nodes: any IoT communication is mediated by MicroELements software components [15], which are instantiated within a first-mile edge node’s network and that can be assimilated to Virtual Machines handling transmission requests of IoT devices towards the cloud. First-mile edge nodes can have multiple MELs, which can aid with load-balancing. Within this work, we compare the effectiveness of different MEL-allocation policies suitable for a 6G, cell-free environment to determine which best optimises the resource utilisation of the MELs within the first-mile edge nodes.

Similarly to the cell-free-based architectures, the original IoTSim-Osmosis-RES architecture [18], the base of SimulatorBridger [13], has a Central Agent controller connected to each first-mile edge node within each subnetwork while monitoring the global state of resource utilisation, as well as routing data flowing through dedicated communication channels. The present paper enhances the former strategy by also supporting the redirection of the packets from the nearest first-mile edge node directly accessible by an IoT device to a “quieter” one belonging to a non-bottlenecked communication network. In our previous network topology [13], the only first-mile edge nodes that could be chosen for a given IoT transmission were the ones in which the IoT device was located. So, if a particular edge subnetwork handled many IoT device communications, the Central Agent could not have used the resources from a quieter subnetwork exhibiting less communications. In the present architecture, packets can be redirected from one edge to the other via a dedicated ring network [49], only connecting all the first-mile edge nodes and being used for re-dispatching the packets across different edge subnetworks. This enables the Central Agent’s selection of any first-mile edge node as the mediator of the communication towards the cloud as initiated by the IoT device, thus increasing the pool of available network resources the Central Agent has at its disposal.

To streamline the packet forwarding and not re-duplicate communication resources, the ring network does not directly connect the first-mile edge nodes with the cloud data centre or the other nodes within each edge network (the gateway and the switch nodes), while still requiring that all the edge nodes belong to the same city and that all the edge networks belong to the same metropolitan area network (MAN). Given that data centre nodes are the root nodes for a edge network [48] where the intermediate nodes are switches, the ring network only connects a minority of the nodes, with the full communication transfer towards the cloud still requiring data transmission via the vast majority of the network nodes.

### 3.4. Simulation Output

SimulatorBridger utilises load-balancing algorithms, such as round-robin, enhancing its ability to handle high device density in emergency healthcare settings. The generator includes a module that simulates and analyses IoT device density and battery consumption, illustrated in Figure 2, providing insights into energy usage across various healthcare scenarios. Power management is critical for IoT-based healthcare systems, particularly in continuous monitoring, where devices must remain functional over extended periods. Studies highlight the importance of secure, energy-efficient data transmission to protect sensitive health data while maintaining battery life [50,51]. In this paper, we limit the study of the simulator to the sole processing time and number of connections active per second. Still, all the aforementioned information is still collected and stored within our relational database (Figure 3).

## 4. Packet Routing and Management

To address the challenges of processing healthcare data in real time, the generator includes a simulation of cell-free architecture built on top of the pre-existing computing environments considering a Central Agent connected to the data centres governing each edge network [13]. It models the interactions between mobile IoT devices, those on ambulances, and static first-mile edge nodes, RSUs, ensuring that data can be processed and transmitted efficiently even under varying network conditions. The generator also incorporates elements of Osmotic computing such as MELs. The generator provides insights into optimising data processing in healthcare scenarios by simulating different MEL-allocation policies and their impact on communication times.

Within SimulatorOrchestrator, the Central Agent now has knowledge of both the processing capabilities of the MELs, their current processing load, and the SDN bandwidth allocations of all of the communication channels within the network. Using these data, the Central Agent is then given the power to choose which MELs an IoT device communicates with and, depending on the routing algorithm chosen, which MEL within the network processes a given communication. In our previous solution [1,22], a round-robin policy decided which MEL would initiate the communication to the cloud for the IoT device; this same MEL was also responsible for processing the communication incoming from the IoT device before forwarding it to the cloud; the round-robin policy only selected from the edge’s MELs within the nearest edge infrastructure to the IoT device initiating the communication. To enable SimulatorOrchestrator for the first time to simulate environments compliant with the unravelling 6G standard requiring more connectivity and smart solutions for ensuring QoS, we extended the Central Agent to both monitor and control the entirety of the network, as well as instructing the MEL receiving the packet from the IoT device to re-forward their packet via the token ring if a better edge MEL was found to initiate the communication with the cloud. This ultimately extends the previous SD-WAN protocol; while previously the main edge gateway was solely responsible for the packet-routing strategy, a more interconnected network also enables the Central Agent to make decisions. The present paper will show that such a decision will result in more efficient packet transmission, enabling faster communication transfer, which will be crucial in emergencies or life-threatening situations.

### 4.1. Dynamic Packet Routing for Edge Sub-Networks

As discussed in Section 3.3.3, the edge networks are managed by gateways being data centres with SDN controllers: as such, they route the transmission from the IoT devices (e.g., vehicles) to the backbone network, which is connected to the cloud data centres. Software-Defined Networking (SDN) allows for the precise management of network traffic flow within IoT city infrastructure by the SDN controllers through software. These SDN controllers can control the different areas within a network’s topology, such as its network switches, allowing it to decide how best to manage a network’s resources [13]. Given both the SD-WAN and SDN cell-free controllers use software to optimise the routing of the transmissions up to the cloud data centre, different routing algorithms can be used to control the path these transmissions take. Within this work, we compare two different routing algorithms: Shortest Path Maximum Bandwidth (SPMB) [17], being an extension of Dijkstra’s shortest path algorithm [52], and Minimum Cost Flow Routing (MCFR) [22], based on a multi-source and multi-target extension the minimum cost flow problem [53], and their impact on network delay within the cell-free architecture.

SPMB is a modified shortest path Dijkstra algorithm that aims to find the shortest path for each transmission toward the cloud data centre with the minimum number of traversing network nodes. If multiple shortest paths exist, the one with the highest available bandwidth is selected. The pseudocode for this algorithm can be found in [17].

MCFR [22] maps latency minimisation as a multi-source, multi-target minimum cost flow problem. The latency between two network nodes is set to be directly proportional to the distance between the two nodes. The sources for this algorithm are the communicating IoT devices, VMs in the destination cloud data centre are the targets, and the flow is the number of communicating IoT devices. This defines a network in which both IoT devices and MELs can act as sources communicating with target MELs, provided they are within the communication radius of the target MELs, and a channel capacity has an upper bound of the number of communications the target MELs can currently handle.

### 4.2. Packet Limiter

Regardless of the active routing algorithm, we consider a packet limiter strategy [22], effectively limiting how many active communications each MEL can have with the cloud data centre. MELs consider communications active until the cloud data centre has processed the communication and notified the IoT device that the communication is “finished”. The limiter algorithm is set to a threshold of three communications active between each MEL and the cloud data centre; all other communications which arrive at each MEL are held in a waiting queue on the MEL until an active communication finishes and a new communication can be sent from this MEL.

### 4.3. MEL “Quietest” Allocation Policies

The goal of our MEL-allocation policies is to aid in balancing the network load. We could send all the network traffic to one MEL, but this would bottleneck this MEL, and all the other MELs would be wasted resources. The best MEL policies aim to maximise the efficiency of the network, utilising as much of the network’s transmission capabilities at all times when transferring communications from the IoT devices to the cloud. Thus, we want to seek the “quietest” (i.e., the least busy) MELs within the network to mediate the communication with the cloud.

The Central Agent and CPU within the cell-free architecture dictates which MEL initiates the transmission on behalf of the IoT device after the MEL receives the request from the IoT device. The same MEL performed these two actions within SimulatorBridger. Still, as SimulatorOrchestrator now contains a ring network solely connecting the first-mile edge nodes containing the MELs, we can decouple these two processes from the same MEL, thus obtaining better transmission balancing. We are modelling this ring network as instantaneously transmitting communications between MELs, as we are focussed on how the network between the MELs and the cloud is affected by this new architecture, and this transmission time along the ring network will be negligible compared to the overall transmission time. This decision comes with the obvious cost of additional realism; most importantly, bottlenecks can arise within the ring network itself [54], which we are not modelling with our idealised instantaneous transmissions. As per the previous simulator, IoT devices are simulated to only transmit to the MEL within their communication radius [13].

The ring network enables the Central Agent/CPU to choose the optimal MELs to process incoming communication: this boils down to the definition of novel MEL-allocation policies beyond the simplistic MEL selection among the ones directly available through the ones reachable from the first-mile edge node appearing in the first-mile communication with the IoT device. The choice between the two routing algorithms, SPMB and MCFR, also affects the path a communication might take through the SDN-SDWAN architecture, as these two algorithms differ on both how and when the decision is made in deciding the path a communication will follow through the network. Therefore, we have different MEL-allocation policies for the other routing algorithms.

#### 4.3.1. MEL Quietest Allocation for SPMB

The SPMB algorithm reassigns communications at the beginning of the IoT transmission time. Then, the Central Agent checks for the best MEL to initiate the communication on behalf of the IoT device, at which point it is sent along the ring network to that MEL.

Algorithm 1 uses information on the MIPS (million instructions per second) available to each MEL to make informed decisions of which MEL is spending less resources at transmission time. Without further information, communications are initially assigned to the MEL the IoT device communicates with by default (currentMEL, Line 8). Before starting the processing of the information, it is reallocated to a quieter MEL when possible (Line 7) and sent along the ring network to the quieter MEL. When a MEL is given a task, such as processing a communication, it uses some of its computational power while depleting the remaining available resources. When the Central Agent decides which MEL to allocate a new job to, it selects the MEL with the most available MIPS (Line 5). In the case of multiple options, it arbitrarily selects the MEL first yielded by the for loop (Line 4). Given that the MIPS values are not updated until the task is started, in the event of a batch of communications, the Central Agent may have to allocate multiple MELs before the MELs can begin their transmission and then actually start consuming MIPS resources; in the worst case scenario, this would lead to allocating the entire batch for one same MEL.To prevent this, the MIPS value for that MEL is subtracted by 110. We decided upon this value as, throughout preliminary experiments, it produced the best transmission times but is otherwise arbitrary. Additionally, if the MIPS value is below 0.1, the subtracted amount changes to 0.1 multiplied by the current value (Line 6). This, overall, leads to decreasing the priority of a given MEL directly proportionally to the time this was originally picked.
**Algorithm 1** MIPS-based Quietest Allocation for SPMB (SPMB Quietest)**Require:**     melMIPS: a function mapping each MEL to their current remaining MIPS value1:**function** SPMBQuietest(currentMEL)2:    M←maxcod(melMIPS)                     ▹ *The available MIPS on the least busy MEL*3:    μ←mincod(melMIPS)                           ▹ *The available MIPS on the busiest MEL*4:    **for all** MEL **in** dom(melMIPS) **do**5:        **if** melMIPS(MEL) =M **then**6:               melMIPS(MEL) ←max(M−μ·110,M−110)      ▹ *Update melMIPS value*7:               **return** MEL                                                      ▹ *Choosing the quietest MEL*8:    **return** currentMEL

#### 4.3.2. MEL Quietest Allocations for MCFR

When the MCFR algorithm is used, the MEL allocation of the policy outlined above for SPMB is no longer applicable. While SPMB makes an earlier decision regarding which MEL will start processing on behalf of the IoT device, our MCFR implementation reassigns the MELs as soon as they arrive at the initial MELs by design and before any communication starts and, therefore, before any MIPS usage can be updated.

This means if any MCFR Quietest policies attempt to allocate MELs based on the MELs’ relative current workloads, they would be doing so with outdated information. To remedy this issue, whilst still aiming to maximise the efficiency of the network, we made improvements to the two algorithms found in [1]; namely, round-robin allocation policy per MEL and round-robin allocation policy per IoT Device, utilising the additional functionality provided by the ring network. For quiet first-mile edge nodes with quiet MELs, the network should not be impacted based on the preferred quietest MEL.

Despite the addition of the ring network, the fundamental behaviour of the two allocation policies is the same. The first round-robin policy, ${\mathrm{Quietest}}_{1}$, sends each *i*-th communication to the *i*-th MEL until each MEL has been allocated a communication, at which point the algorithm returns to the first MEL. Similarly, the round-robin policy per IoT device ${\mathrm{Quietest}}_{2}$ results in the *i*-th communication from each device being sent to the *i*-th MEL. This can be achieved by simply changing the location of the data structures and keeping track of the MEL allocation. While a global counter allows the former to be achieved, keeping track of the allocation per IoT device enables the second protocol to be achieved. This approach is summarised in Algorithm 2. The only exception to this behaviour is when the Central Agent must simultaneously reallocate MELs for multiple communications. For a batch of size *N*, the Central Agent will allocate *N* MELs, or all MELs if *N* is larger than the total number of MELs, for this batch. The communications will then be allocated to the MELs closest to their originating device.
**Algorithm 2** MCFR MEL-Allocation Policies
  G denotes a graph of a function

  ${\mathrm{Quietest}}_{1}$: #edgeNet and MELsCount are updated globally

  ${\mathrm{Quietest}}_{2}$: #edgeNet and MELsCount are updated locally per IoT device**Require:**
  δ: physical distance function between physical devices (IoT, MELs in first-mile edge nodes)

  ls: associates each IoT device with the current Nearest MEL

  #edgeNet: associates each edge network with the number of times being chosen

  MELsCount: associates each MEL with the number of times being chosen

  e2M: defines MELs belonging to a single-edge network

  thisLoop: counting the number a MEL is chosen at each iteration
 1:**function** RoundRobin(#edgeNet, MELsCount, ls)⊳ Quiet MEL selection and count update 2:      $\mathrm{quietNets}\leftarrow \left\{\;net\in \mathrm{dom}(\#edgeNet)\;|\;\#edgeNet(net)=\min\mathrm{cod}(\#edgeNet)\;\right\}$ 3:      $G\left(\mathrm{quietMELs}\right)\leftarrow \left\{\;\left(m,\mathrm{MELsCount}(m)\right)\;|\;m\in \mathrm{dom}(\mathrm{quietNets}),e2M(m)\in \mathrm{quietNets}\;\right\}$ 4:      **for all** MEL **in** $\arg\min_{m}\mathrm{MELsCount}\left(m\right)$ **do** 5:            *MELsCount*(MEL) ←MELsCount(MEL)+1 6:            *thisLoop*(MEL) ←thisLoop(MEL)+1 7:      **for all** MEL **in** dom(MELsCount) **do** 8:            #edgeNet(e2M(MEL)) ← #edgeNet(e2M(MEL)) + *thisLoop*(MEL) 9:      G(thisLoop)←∅ ⊳ IoT-MEL re-mapping over the quiet MELs10:      **return** $\left\{\;\left(\mathrm{iot},\arg\min_{\mathrm{mel}\in \mathrm{dom}(\mathrm{quietMELs})}\delta \left(\mathrm{iot},\mathrm{mel}\right)\right)\;|\;(\mathrm{iot},m^{\prime })\in \mathrm{ls}.\;\right\}$

As previously mentioned, first-mile edge nodes are hosts within edge network data centres controlled by SDN controllers. If there are no communicating IoT devices near a potential host, then that host is inactive, which means some edge networks have more hosts and, therefore, more MELs than others. If allocation policies do not consider this fact, then simply evenly distributing the network load across the MELs results in some edge networks needing to route more communications towards the backbone gateway than others. The updated round-robin algorithms account for this by choosing the edge network via round-robin and then choosing a MEL on that edge network via round-robin.

## 5. Experimental Analysis

We now outline our experiments comparing the network performance of cell-free 6G architecture generalising over a pre-existing cellular 5G architecture: the former enables the Quietest MEL allocation strategy while the latter only encompasses the usage of the Nearest one within the same network. The 5G infrastructure also utilises the round-robin per MEL allocation policy [1], and both architectures have a network latency of 1 ms and the bandwidth between the IoT devices and the cloud is 10 Gbit/s [55]. The vehicles transmit communication towards a nearby edge node every 75 ms, provided they are within the communication radius of an Edge, which still simulates 5G Edge nodes. As outlined in Section 4.3, different MEL-allocation policies are implemented depending on the chosen routing algorithm. Within SimulatorOrchestrator, we change the architecture by deciding whether a MEL processes communications from an IoT device within the edge network local to the IoT device, 5G, or if they can be transferred to a more optimal, quiet MEL by the Central Agent along the ring network, 6G. We will refer to these architectures as ‘Nearest’, for the 5G architecture and ‘Quietest’ for the 6G cell-free architecture and accompanying MEL allocation policy, outlined in Section 4.3. Given that there are two MEL-allocation policies for MCFR, the round-robin per MEL and round-robin per IoT device will be referred to as ${\mathrm{Quietest}}_{1}$ and ${\mathrm{Quietest}}_{2}$, respectively.

### 5.1. Experimental Setup

We ensure the initiation of IoT traffic data within the first 300 s of simulation time as determined by the SUMO vehicles passing near RSU, thus initiating the communication with the cloud, including vehicles equipped with IoT sensors and patients transmitting their health data, both towards a cloud data centre via the edge. To generate the vehicle traffic, we exploited mobility traces from a Bologna Andrea Costa scenario (Section 1). These mobility traces contain vehicles driving on roads in a 2 km by 1 km area near a street named ‘Andrea Costa’ within a scenario aiming to simulate the expected vehicle traffic, which occurs as a result of ‘big events such as football matches or concerts’, whilst also being situated in the nearby vicinity of a hospital [56,57]. The municipality of Bologna provided the necessary data for the road networks while confirming the SUMO provided a realistic simulation of urban traffic patterns. These considered configurations such as traffic light placements, vehicular departure times, and vehicular routes [56]. This dataset contains two route files, one for cars and another for bus lanes; we only use car traffic for this analysis (709 vehicles within the first 300 s). The position of the RSUs within the network leads to the generation of the edge subnetworks via the placement of the first-mile edge nodes, from which the remaining communication nodes are built upon. Following the architecture laid out in Figure 1, Table 1 shows a breakdown of the specifics of the network used within our experiments: edge networks exhibit a variable number of first mile hosts, with varying numbers depending on the RSU locations (from 4 to 1). Finally, we consider both the limiter algorithm and the network packet routing algorithms such as SPMB and MCFR (Section 4), where the former limits each MEL to three active communications between itself and the cloud, as in [22]. After running the simulator, we collect all the logged information within our relational database and analyse the observed simulated behaviour.

As per Section 1, we place 60 patients in the environment: 3 at 15 of the 16 first-mile edge nodes, modelling home cared patients with different seriousness levels of health conditions, and 15 at the 16th first-mile edge node to model a hospital. IoT devices were placed at each edge node to model patients within the smart environment. As shown in [1], it is sufficient to know only how many communications are received at the edge nodes to model the overall network’s behaviour accurately. Therefore, we do not need accurate patient position data, only which edge node they transmitted their health data towards. Patients, whilst healthy, transmit their data every 10 s, which changes to every 1 s when their health data determine they are an at-risk patient. To generate the patient data, we used our own patient digital twin generator (Section 3.3.2), within which patients are assigned as low risk, medium risk, or high risk depending on their given initial blood pressure, heart rate, and core body temperature mean and standard deviation (σ) values. Table 2 shows the initial values for the patients at each risk level and the healthy ranges for each metric, as per Section 3.3.2. These values are based on the current literature. If any of the three metrics deviate from the healthy range, the ’health risk score’ of the patient increases (see Section 3.3.2), and once the patient’s health risk score reaches 3, the patient becomes a high risk and transmits their data towards the cloud more frequently.

### 5.2. Active Communications (ACs) and Processing Times on the Edge

**Figure 4:**
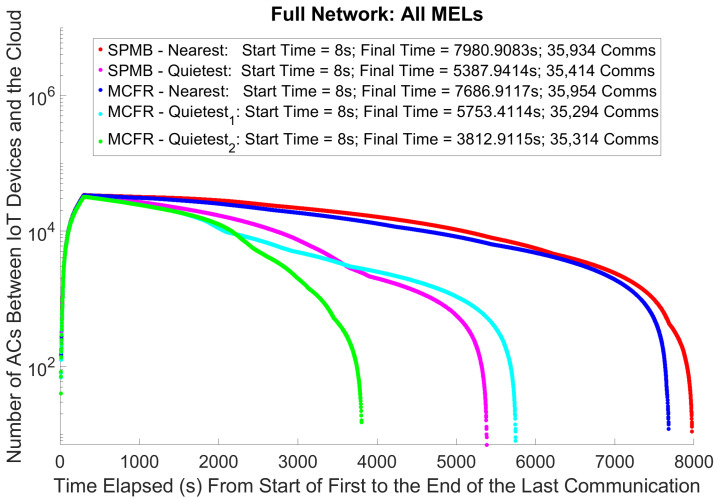
Visualising the start times, end times, and number of active communications (ACs) for each of the 5 runs comparing the Nearest and Quietest architectures for both SPMB and MCFR.

**Figure 5:**
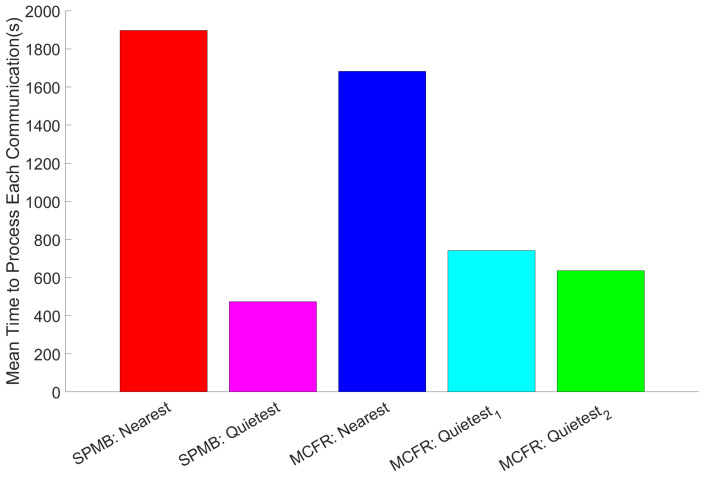
Visualising the impact of both the routing algorithm and network architecture on the edge processing times.

We are interested in how the 6G architecture reacts to the overall transmission of communications from IoT devices to the cloud via the edge: Figure 4 considers communications active from the moment the IoT device transmits them. The most obvious result from Figure 4 is that the 6G cell-free architecture does improve the network’s performance. This shows that even the slowest quietest run, MCFR ${\mathrm{Quietest}}_{1}$, is over 1900 s faster than the fastest Nearest run, MCFR Nearest. This result aligns with expectations: communications are processed faster as routing communications towards the MELs with the current lowest workload, which can seen in Figure 5, and therefore transmitted towards the cloud earlier, lowering the overall time taken for communication to both reach the cloud, which allows the cloud to start processing communications earlier, which in turn helps to lower the total time for communications to “finish”. Also aligning with expectations is SPMB quietest; we achieve lower final time than SPMB Nearest. SPMB Quietest exploits the Central Agent from the cell-free architecture with the information on the current MIPS available for a new job, allowing the Central Agent to assign jobs to the MELs with the highest available MEL. This is in contrast to the Nearest implementation, where communications are sent to the nearest edge node, and then assigned to a MEL in a round-robin fashion. If the edge node is in a busy area, all MELs will be busy/bottlenecked, so all communications originating in this area are sent to bottlenecked MELs. Thus, it takes longer for both communications, and therefore, the packets are transmitted towards the cloud later. The overall time to transfer communications from the IoT devices to the cloud takes longer, resulting in a higher final time.

First and foremost, the 6G cell-free architecture has significantly improved the network’s performance. The cell-free architecture, with the addition of the Central Agent and ring network, allowed for improved MEL-allocation policies to select MELs based on which were best suited in real time to transmit communication towards the cloud, no longer being constrained to selecting MELs based on their physical locations within the environment. These architectural improvements aim to reduce the overall time required to transmit all the communications from the IoT devices to the cloud. Using the start and final times from Figure 4, we can obtain the overall time for all the communications to reach the cloud under each configuration. Comparing the overall times for the Nearest and Quietest MEL-allocation policies for each of the two different routing algorithms shows that these architectural improvements resulted in a 32.49% reduction in the time taken to transmit all the communications for SPMB and a 25.15% and a 50.4% reduction for MCFR ${\mathrm{Quietest}}_{1}$ and ${\mathrm{Quietest}}_{2}$ policies, respectively. MCFR performed better under the 5G cellular configuration, ‘Nearest’ configurations on Figure 4, than SPMB, suggesting that it can efficiently route the communications through the network even under pre-existing 5G technologies.

Although MCFR ${\mathrm{Quietest}}_{2}$ results in the shortest overall transmission time for communications from IoT devices to the cloud data centre, the SPMB Quietest MIPS-based algorithm, Section 4.3.1, results in the lowest average edge processing time per communication, Figure 5). This aligns with expectations, as the SPMB Quietest MIPS-based algorithm specifically looks in real time at the available MIPS on each MEL and chooses the MEL with the most available MIPS when needed. In contrast, the MCFR Quietest algorithms look to balance the workload across the MELs, expecting that this will result in the MELs evenly sharing the total processing workload and preventing bottlenecks. In short, the SPMB chooses the MEL specifically best suited to process a given communication; the MCFR algorithms distribute the workload across the MELs to prevent the edge networks from becoming overloaded.

As a result of the introduction of a cell-free 6G architecture, we observe that the previous results on the 5G without token ring and Central Agent involvement (SPMB Nearest and MCFR Nearest) cannot be transferred to this novel solution. We initially showed in [1] that both MCD (minimum common denominator) [1] and MT (mobility trace) data behave the same under specific 5G settings. Now, 6G prefers MT data: as per Section 4.3.2, we are now favouring re-routing a packet to be transmitted from a less busy edge sub-network rather than forcing the choice only between MELs belonging to the same Edge network. Adding the ring network and orchestrated re-routing mediated by the Central Agent enables better load balancing, thus helping to decongest trafficked networks.

From Figure 4, each of the five runs resulted in 35,000–36,000 communications, although each run should be subject to the same network load from the IoT devices, both vehicle and patient. This results from different MELs being available at different times depending on their assigned workload, which depends on both the routing algorithm and the MEL-allocation policies. A crude way to account for this variation is to divide the total number of communications by the total time taken to transfer all the communications toward the cloud, and final times subtract start times from Figure 4. Table 3 shows that, by doing this, we obtain the same trend as simply looking at the final times: MCFR ${\mathrm{Quietest}}_{2}$, round-robin per IoT device, is the fastest to transfer communications to the cloud, and SPMB Nearest is the slowest. This means we can discount this variance for this analysis, and future work can determine how consequential the lower communication number is to any data analysis performed by a smart city.

### 5.3. Cloud Processing Times

**Figure 6:**
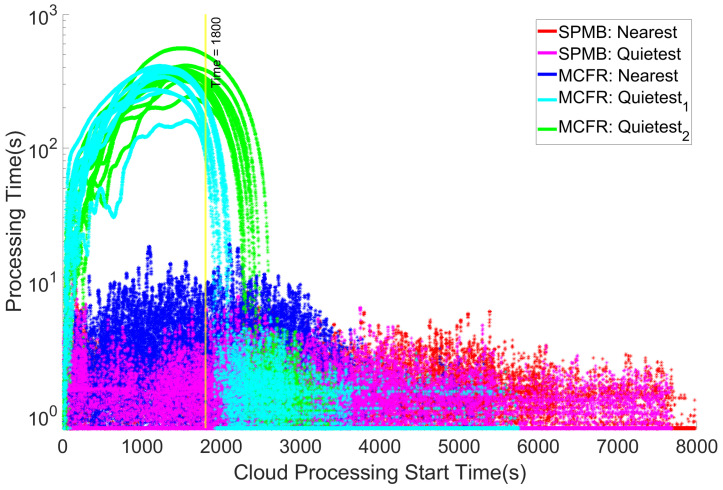
The impact of routing algorithm over a specific architecture on the cloud processing times.

The results so far have focussed on the MEL processing and how the different architectures affect the MEL processing of the communications, impacting the final simulation times shown in Figure 4. However, as was seen in [22], if many communications start reaching the cloud too quickly, processing times on the cloud will increase, as its resources are split among the virtual machines processing the data provided by all incoming communications. As per Figure 6, both MCFR Quietest configurations cause the cloud processing times to spike early in the simulation. This is because the edge nodes with these configurations process communications more quickly than the Nearest configurations, transmitting communications towards the cloud more quickly. Whilst this does mean more communications reach the cloud in a shorter period, it also results in communications reaching the cloud more rapidly than the cloud can process its current communications. This leads to the number of communications concurrently processing at the cloud spike, which causes an increase in the time taken to process the communications in their entirety.

### 5.4. Temporal Distance Between the Start of the First Communication and End of the Last One

**Figure 7:**
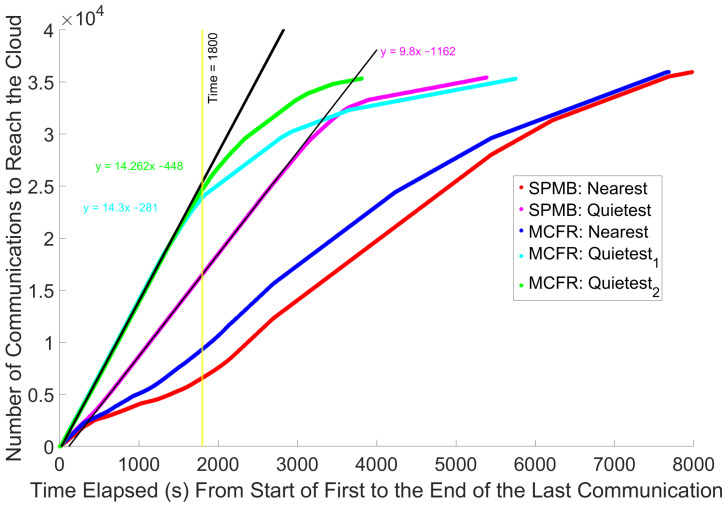
Visualising the rate at which the different algorithms transfer communications from the IoT devices to the cloud. The y-axis represents the number of communications transferred until the simulated time on the x-axis. The black lines show the gradients corresponding to the equations.

Figure 7 shows the number of total communications reaching the cloud throughout the simulation for each algorithm. Consistent with Figure 4, all the communications reach the cloud earlier when using the Quietest policies, compared to the Nearest policies. By comparing Figure 6 with Figure 7, we can observe a correlation between the initial spikes to the cloud processing for the two MCFR Quietest algorithms observed in Figure 6 and the rate at which communications reach the cloud from the MELs when using these two algorithms. Both algorithms can sustain a rate of ≈14.3 communications per second between the MELs and the cloud for the first 1800 simulated seconds (yellow line in both Figure 6 and Figure 7), which correlates with the time for the high cloud processing times. After this mark, both algorithms evidence a transmission rate drop, when the cloud processing times also begin to drop. The MCFR ${\mathrm{Quietest}}_{1}$ transfer rate drops lower and faster in Figure 7, which correlates with a faster drop in the cloud processing times, as shown in Figure 6. Finally, the SPMB Quietest algorithm transfers communications to the cloud more slowly than both the MCFR algorithms, ≈9.8 communications per second as per the magneta slope in Figure 7. This communication transfer rate does not lead to a spike in cloud processing times as per the magenta in Figure 6, suggesting the cloud is able to process communications faster than 9.8 communications per second, but not at 14.3 communications per second.

This suggests a relationship between the communication transfer rate and the cloud processing times. Given that Figure 7 shows that the communication transfer rate always decreases for each of the five algorithms, this drop in communication transfer rate is unlikely to be due to the algorithms or the behaviour of the MELs. On the contrary, this most likely only occurs once the total number of communications remaining to be transmitted also drops, and fewer communications are available overall to transmit. Each MEL has fewer communications to transmit and can no longer operate at full capacity. MELs have many communications to transmit, and they can do so quickly as a combination of the processing improvements brought by the Central Agent and ring network coupled with the improved routing of communications through the network from the MCFR algorithm. However, this increased transmission rate by the MELs also increases the rate at which communications reach the cloud. From Figure 6, this rate is shown to be too high when using the MCFR Quietest policies for the cloud to process its current workload before receiving new communications, as the MELs can maintain the transfer rate of ≈14.3 communications per second toward the cloud (Figure 7). The cloud processing only recovers once the rate of communication transfer slows. This also suggests the limiter algorithm [22] can no longer adequately limit the rate at which communications reach the cloud, leading to the return of the cloud processing bottlenecks addressed within [22].

### 5.5. Scalability Analysis

The results in the above analysis were generated from 300 s of IoT traffic generated from SUMO; in this, any vehicle initiates the communication when approaching a first-mile edge Node. This leads to communications being initiated by the IoT devices, while the transmission of the packet to the cloud is mediated and handled by the Edge Node. Contextually, patients share the first-mile edge nodes with the vehicular traffic. These communications were routed from the edge nodes through the network towards the cloud hosts in the cloud data centre (see ‘C’ from Figure 1). While doing so, the simulator keeps track of the evolution of each agent within the simulation while also tracking potential changes to the packet routing as a consequence of an increase in the overall network traffic.

Table 4 shows both the simulated time generated needed to route all the communications from the IoT vehicles to the cloud (ST), the real-world time needed to simulate this communication transfer (RWR), and the difference between the two (DIF), always in the context of the same experiments as per the previous sections. DIF values heavily depend on the algorithm used. This is an expected behaviour, as each MEL allocation policy impacts the simulation time differently. Our experiments remark that a more efficient packet-routing strategy also comes at the cost of increased time complexity for determining the best routing and re-routing of the undergoing communications within the SDN, thus significantly impacting the simulation time. SPMB Nearest is over an hour faster in real time despite requiring to simulate 5 extra minutes before scheduling the Cloud shutdown than MCFR Nearest. While SPMB uses a variant of the shortest path algorithm, MCFR uses a variant of the maximum flow minimum cost problem associated with greater time complexity. Furthermore, the MCFR ${\mathrm{Quietest}}_{2}$ MEL allocation policy is further aggravated to track which MEL each originating vehicle has or has not communicated with. This further builds up the time complexity with additional overhead. On the other hand, this policy results in the lowest final time to transfer all communications from the IoT devices to the cloud, so SimulatorOrchestrator needs to simulate less time, which lowers the runtime.

To gauge the scalability of SimulatorOrchestrator more accurately, the MEL allocation policy needs to be kept constant whilst varying other network factors, such as the number of IoT devices within the simulation. Given that MCFR ${\mathrm{Quietest}}_{2}$ results in the lowest simulated time, the scalability of this algorithm is of the most interest, and thus will be used in the present analysis. This is conducted by gradually removing vehicles from the Andrea Costa dataset while keeping the number of patients constant. This enables us to determine the impact of the number of IoT devices on the simulation runtimes. Given the overall 709 different vehicle entries within the dataset within the SUMO simulation times 0–299, we removed 1/6 of the total vehicles (or 118) at a time to see how the real-world time of the simulator was affected (Table 5, part A). To ensure a similar distribution of the vehicles entering the simulation as the overall dataset, we performed a uniform sampling over the birth distribution of the vehicles entering the simulation by considering a binning of 10 s. As vehicles communicate with RSU, acting as first-mile edge nodes, vehicle distribution changes might still lead to unexpected behaviour, albeit trying to maintain a similar vehicular birth process. For example, removing a vehicle at the front of a traffic jam might allow one further back to be in the range of an RSU, keeping the total number of communications the same. This information cannot be easily reconstructed statically, thus requiring complete node information. Due to the high volume of communications (34,000–36,000) resulting from traffic initiated within the first 300 s of simulated time, all the communication data are stored within a relational database (Figure 3). This allows for the detailed post-mortem analysis discussed earlier, which further contributes to the increased runtime of the simulation. This solution became necessary as the JSON storage solution from [13] ran into primary memory issues as the generated data from the simulator exceeded 32 GB of JSON data. The overhead caused by a Relational Database is particularly relevant when injecting the patient data, as this needs to be conducted during the simulation. In contrast, vehicular data were stored and never updated at the beginning of the simulation (Figure 3). Therefore, we keep the number of patients constant and only vary the vehicle number to accurately determine if the number of IoT devices impacts the simulation runtime.

From Table 5 part A, lowering the number of IoT devices decreases the simulator runtime, and for 5 out of 6 of the runs whilst simulating a similar time of ≈1 h. For the run with only 118 vehicles and 60 patients, there is a sharp drop in the simulator runtime, as it simulates 46 min in just under 4 min of real time. This is most likely due to a sharp drop in the interactions to be modelled within the scenario, as we handle ≈1/3 of active communications if compared to the same situation where all originally simulated vehicles are present. Additionally, the decrease in vehicles is clearly directly correlated with the decrease in complex interactions occurring between communicating agents within the simulation, which overall led to a more efficient simulation. As reducing the number of vehicles also decreases the number of total communications, which might not be necessarily related to the number of IoT devices considered within the simulation, this ultimately remarks that the simulator is heavily affected by the number of communications to be modelled. To investigate this, we re-ran SimulatorOrchestrator by keeping the maximum number of vehicles while varying over the types of patients. In particular, we only consider 20 patients per run while using only the patients from one specific risk group. Part B from Table 5 solidifies the former consideration: as the number of communications increases due to the increased health risk requiring streaming more communications to the cloud, so does the simulation runtime. By comparing the results from Part A with the ones from Part B, all three results from the latest experiment have lower real-world runtimes than the 472 vehicles run from the earlier experiment, despite Part B having more initiated communications. This discrepancy is likely due to the number of closed communications and the additional patient devices’ simulation leading to new communication events stored within the relational database as timedIoTs.

By keeping the maximum number of vehicles and removing all the patients (Table 5 part C), we obtained an ST time very similar to the scenario in Part A, where an additional 60 patients were considered, notwithstanding that we now only run the simulator for 18 min. By further increasing the number of total IoT vehicular devices by generating more vehicular traces and extending SUMO so to generate communication events initiated within the first 600 s of the simulation (Table 5 part D), the simulator dealt with almost double the number of communications in a comparable amount of real-world time to the former scenario. This might suggest that the streaming of patient data leading to a heavy relational database restructuring might be responsible for the increase in simulation runtimes. DIF values tell us that the significant simulator overhead is caused by simulating an increased number of active communications within a single time frame. The drop of DIF from Part C to Part D also suggests that, if we let the simulator deal with an increased number of vehicular communications scattered throughout a larger time frame, we will potentially end up with RWR becoming larger than ST time, as previously evidenced MCFR ${\mathrm{Quietest}}_{2}$ within the former experimental setting (Table 4). These results suggest that there is no simple solution to further improve SimulatorOrchestrator’s scalability, as the number of vehicles, the number of communications, the type of input data, and the complexity of the simulated scenario all contribute to increasing the time required to carry out the simulation. In fact, the nature of the routing algorithms requires us to model our simulator as a sequential multi-agent representation of a communication network, thus entailing that any increase in the simulator complexity will naturally lead to a rise in simulator runtime.

## 6. Discussion

The simulator simulates 300 s of vehicular mobility, generating 34,000–36,000 communications, resulting in 3800–8000 s (i.e., ≈1H to ≈2H and 30 min) of overall simulated time (see Table 4) for transferring the data packets from the IoT devices or patients to the cloud via the entire communication infrastructure. The real time needed to simulate these runs varied quite widely (Section 5.5). Such variation ultimately depends on the architecture (5G or 6G), its Osmotic configuration, and the routing algorithm. This relatively long simulator runtime, ≈1 h for the Quietest configurations, also justifies our simulator accounting for just the Presentation and Session layers of the OSI stack; despite the fact that we simulated thousands of seconds of network behaviour, it still is only a consequence of IoT network traffic generated within the first 300 s of the simulation. Due to this, wireless communication simulation will not account for real-world network behaviours such as contention [58]; notwithstanding the former, our simulator is consistent with previous network simulators’ assumptions in this domain, simulating only the required OSI layers for this work (Section 2.2), particularly IoTSim-Osmosis-RES [18], which was the basis for our initial SimulatorBridger work. This decision was taken because the original IoTSim-Osmosis-RES simulator also works on SD-WAN networks, thus justifying our modelling choice.

In our previous work [1], the connection-counting approach mapped each counted communication to a fresh IoT device at a given time. Using communication counting for healthcare devices associated with patients required a more precise mapping, which was then revised in the current pipeline. As a result, the round-robin per IoT device policy would send all communications in a given area to the same MEL, as all the counted devices communicated only once. As we now possess the information of which device originates the communication in the form of the patient ID from the patient sending the data and vehicle ID from the mobility traces, we can better simulate the overall network behaviour, thus resulting in a more faithful simulation of the round-robin per IoT device policy.

The SPMB algorithm did not result in the overall fastest total transmission time despite resulting in the best average edge processing times per communication, Table 3), suggesting that the MCFR algorithm is better suited for routing a realistic network load from the IoT devices to the cloud. In [22], both SPMB and MCFR were stress tested for the 3G, 4G, and 5G cellular networks, and the results showed that the optimal algorithm was dependent on the cellular network in use, although the differences were small. With a more realistic workload in this work, MCFR, compared to SPMB, resulted in a shorter overall transmission time of all the communications from the IoT devices to the cloud data centre. This was true when comparing the two ‘Nearest’ configurations and when comparing the SPMB Quietest with MCFR ${\mathrm{Quietest}}_{2}$. Additionally, despite the faster edge processing rate, compared to ${\mathrm{Quietest}}_{1}$, Table 3, SPMB Quietest, was still slower at transferring communications to the cloud, Figure 7). This can only result from less efficient and slower routing of the communications from the MELs to the cloud. This is a result of the MCFR algorithm aiming to choose the best route for communication through the network, with the smallest latency, and not simply the shortest path, like SPMB.

The fact that the round-robin policy per IoT per device, ${\mathrm{Quietest}}_{2}$, outperformed the round-robin policy per MEL, ${\mathrm{Quietest}}_{1}$ for MCFR may initially seem counter-intuitive as ${\mathrm{Quietest}}_{1}$ should be more evenly distributing the communications across the network. However, due to the specific behaviour of the MCFR algorithm, this is not the case. The MCFR algorithm, when choosing a network link between network nodes within the SDN-controlled data centres, aims to choose the link with the highest bandwidth. The ${\mathrm{Quietest}}_{1}$ algorithm minimises the usage of the specific links between individual hosts and their connected aggregate switches. It maximises the number of links the bandwidth available to the aggregate switches needs to be shared between. This is due to ${\mathrm{Quietest}}_{1}$ causing every MEL, and therefore every edge node/host, to transmit a communication per MEL up the tree network towards their connected aggregate switch, using a share of the bandwidth. The result of MCFR ${\mathrm{Quietest}}_{2}$ is a network saturated with partially used links, each using an equal share of the bandwidth total available to the aggregate switches. MCFR and the round-robin policy per MEL work against each other, slowing the network. In contrast, MCFR ${\mathrm{Quietest}}_{2}$, only uses one link towards the aggregate switches at a time. Each first communication from the vehicle is towards the same MEL on a given edge node/host, meaning the bandwidth available to the aggregate switch can be fully utilised on the link connecting it to that host. The second vehicular communication might then move to a different edge network entirely, keeping these communications from affecting the transmission of the first. MCFR ${\mathrm{Quietest}}_{2}$ is not immune to the network saturation issue affecting MCFR ${\mathrm{Quietest}}_{1}$. In cases of many connecting IoT devices starting the first communication at different times, there will be instances in which multiple hosts within an edge network are simultaneously transmitting towards the same aggregate switch, which will lower the availability of each link. However, this issue is not due to a core aspect of the algorithm, meaning the network will self-correct as the number of simultaneously communicating devices reduces. Additionally, this can be mitigated with MCFR ${\mathrm{Quietest}}_{2}$ by adding more edge networks, as this will allow the number of simultaneously communicating devices to increase before there is a hit to the network. Finally, despite this issue, MCFR ${\mathrm{Quietest}}_{1}$, with this saturation issue, still performs significantly better than the 5G cellular MEL-allocation policies, meaning this is not a disqualifying issue for MCFR ${\mathrm{Quietest}}_{2}$, for which this is a temporary issue which only arises when the network becomes particularly busy. This saturation issue motivates any future work to make additional improvements to the network to obtain the maximal benefit from the MCFR ${\mathrm{Quietest}}_{2}$ algorithm.

To make the most of the MCFR Quietest configurations, particularly ${\mathrm{Quietest}}_{2}$, as this was otherwise the best performing algorithm, further improvements must be made to address these increasing cloud processing times. One solution could be a more proactive approach to MEL selection. The SPMB Quietest algorithm resulted in the lowest average edge processing time per communication (Figure 5), due to the selection of the best MEL in real time to process a given communication based on its available MIPS. The MCFR algorithms could not make use of this data due to the algorithm scheduling routes through the network earlier (Section 4.3.2), and so the MIPS data available at the MEL selection would be out of date by the time a given communication was actually being processed. However, a predictive approach could be implemented, potentially training an A.I. model to select MELs based on the likely workload of the MELs when they process the communication. Combining this approach with the MCFR ${\mathrm{Quietest}}_{2}$ approach to form a weighted round-robin per IoT device, where the algorithm would factor the likely available MIPS, would gain the improved MEL processing from the SPMB Quietest algorithm whilst maintaining the improved routing through the network from the MCFR algorithm. This will also motivate the need for AI to provide greater support in decision-making processes, especially related to learning more flexible routing policies.

Simpler solutions could be implemented: we plan to improve the limiter algorithm. The limiter could be set network-wide, limiting the rate at which communications can be transferred to the cloud to below 14.3 and closer to 9.8 (Figure 7); the limiter could be tuned to rely on bandwidth and not a fixed limit; or the limiter could even be made explicitly time-based, only allowing the transmission of a set number of communications per second. The drawback of these solutions is that they would inevitably slow down the overall transmission of communications from the IoT devices towards the cloud. Therefore, further analysis will also consider other possible ways to improve cloud processing times by calibrating the processing power or potentially adding more cloud data centres for processing the incoming load of IoT data rather than the single one encompassed by both our simulator and previous Osmotic simulators [31]. The benefit of these approaches is that they would not require intentionally slowing down the overall transmission of communications towards the cloud. They would, however, require additional costs and resources if deployed in a real smart city, which tuning the limiter algorithm would not. The best compromise solution most likely encompasses a fine-tuned combination of adding additional computational power/cloud data centres and an improved limiter algorithm.

As outlined in Section 4.3.2, when discussing Quietest policies and with reference to Algorithm 2, when the Central Agent needs to simultaneously reallocate MELs for |ls| communications at the same time, the Central Agent will find the best MELs in *quietMELs* (Line 3). Next, the |ls| communications will be sent to the MELs nearest their originating IoT device (Line 10). However, two issues might occur. First, multiple communications initiated from the same area might end up to the same nearest MEL. As a result of this “multi-reallocation”, there will be MELs from *quietMELs* which do not obtain any communication while others might obtain more than one. This will unnecessarily slow down the processing of some communications as the MELs that receive communications now need to split their processing capabilities across multiple communications. Second, the algorithms keep track of how many times the Central Agent makes a MEL available for allocation, not how many communications are sent along the ring network to each MEL. In the former scenario, all MELs, regardless of how many communications they each receive, will be treated the same moving forward, as if they each received one communication (Lines 5 and 6). In future allocations, some of these MELs will be quieter than the Central Agent thinks, while others will be busier. This can lead to the network’s overall efficiency falling, as the Central Agent’s view of which are the quietest MELs is no longer perfect.

Notwithstanding the issues above, which mainly affect the MCFR Quietest algorithms, when using MCFR ${\mathrm{Quietest}}_{2}$, the total transmission time to route all communications from the IoT devices to the cloud was reduced by 42.35% compared to MCFR Nearest. This was 17.61% faster than the SPMB Quietest; see Figure 4. As such, amending this algorithm to count how many communications are routed along the ring network to a MEL will more accurately track the total connections between each MEL and the cloud. This will distribute the network traffic more effectively and lead to even further reductions in the total transmission time for all communications from the IoT devices to the cloud.

While designing our simulator, we implicitly assumed that all the edge nodes belong to the same municipality area, thus assuming that all the edge nodes will be automatically connected to the token ring. On the other hand, if we assume to simulate different smart cities together, this will automatically connect multiple edge devices to the same token ring. Future works will consider extending the simulator better to isolate edge networks into different and separate token rings; for example, multiple cities could each have their own ring networks and then send their data to the same cloud data centre. This means data still need to be routed from the edge nodes to the cloud data centres; the ring network aids the Central Agent in identifying and selecting the optimal edge node within a smart environment from which to send a given communication. This would also require extending the current simulator to simulate token ring networks’ behaviour and interaction. Additionally, as outlined in Section 2.1, 6G aims to mitigate the location-based issues within cellular networks. As deploying specialised APs for non-urban communications might be costly, we might consider extending our Orchestrator to support satellite-terrestrial networks (STNs), enabling 6G communications [59]. In this regard, Interlink [60] helps reduce the handover delay occurring through their handover scheme Asher. Handover is caused by mobility devices switching satellites as they move in and out of range of different satellites, which are also moving. This is enabled by the usage of a digital twin network (DTN) for better determining the satellite routing and handover scheme across satellites. The same technology might also simulate satellite communications at a WAN or interplanetary scale.

This technology could also have an application to SimulatorOrchestrator, allowing for the simulation of many municipalities, each sending their data towards the cloud whilst keeping track of the vehicles and patients as they traverse the different municipalities. Each municipality could also have a smaller cloud data centre to share its data with a larger centralised data centre. As in the current work, the IoT devices, patients, or vehicle sensors communicate with the edge nodes in urban environments. Still, they would switch to communicating with satellites when not within the core urban areas. The necessary patient and vehicle data would then be routed from the IoT devices towards the cloud data centre via the satellite through dedicated edges. This would require considering the satellite communication simulator as a more advanced version of a digital twin, both accepting communicating events and generating new ones, to route communications across multiple municipalities. The increased number of edge nodes and cloud data centres within the simulated scenario would lead to further simulation overheads, as the additional nodes might generate even more communications.

Last, given the nature of the routing algorithms, our simulator must keep precise track of where the agents are located and whether they intend to start a communication. Given that this simulation embodies the interactions between multiple agents, be they communication nodes or the IoT devices (patients and vehicles), and Central Agents deciding at runtime to re-route the packets and change the communication channels, and given that this simulation is performed sequentially, it becomes evident that an increased number of agents and communications will hamper the scalability of the simulator. This issue can only be partially solved through concurrency, as these solutions could only account for constant factor improvements in the running time, which is still negligible compared to the complex interactions between agents within the simulator. Similar considerations can be addressed by changing the programming language or platform to simulate this. Due to the inherent difficulty of the problem, we believe that the only possible way to improve scalability is to jointly handle communications and strategies while avoiding dealing with one single event separately. Conversely, this solution will deter the realism of the simulation, thus not allowing the tracing of the actual interactions occurring within the simulation. Future works will address novel methodologies addressing the trade-off between simulation realism and scalability.

## 7. Conclusions and Future Works

Variations in the number of active communications between MCD and MT raise critical questions about how different communication strategies, instantiated by multiple or fewer devices, affect cloud data processing times. Understanding how MEL-allocation policies within edge and cloud devices influence communication times is essential. This is particularly relevant when processing medical data on the cloud, where the efficiency of handling vast amounts of sensitive information is crucial. Understanding the relationships between the Osmotic network and the 6G architecture infrastructure is imperative to ensure timely and accurate data processing in healthcare. Exploring further optimal cloud architecture designs in such high-load scenarios could increase processing efficiency and reduce latency, thereby improving the reliability and effectiveness of cloud-based medical services. Understanding how different configurations affect data processing times can lead to improved cloud architectures that enhance the performance and reliability of healthcare applications. As remarked by the latest results on Osmotic architecture [15,31], by optimising the handling of connections and the overall network performance, we can ensure more efficient and effective management of data in Smart City scenarios, ultimately leading to better patient outcomes and more reliable healthcare services.

This paper presents for the first time an extension of an Osmotic simulator for better supporting a 6G architecture by exploiting a cell-free architecture by working upon the Central Agent assumption for orchestrating the Edge networks from our previous paper [13], where there is always a direct link between Edge devices and Central Agent by leveraging the assumptions from the previous literature on Software Defined Network architectures [18]. We simulate cell-free massive MIMO (CF mMIMO) by assuming that all the Edge nodes are also connected in a Gigabit Ring Network, via which the packet is dispatched towards the best Edge device that then, in turn, starts to transmit the packed to the cloud via the entire network infrastructure. Experiments show that the combined provision of a novel SDN routing algorithm first proposed in our previous work [22] alongside the cell-free assumption significantly improves the transmission times of the packets on the network. This preliminary work shows the possibility of improving the 5G architecture solutions to meet the 6G technology criteria of ultra-low latency, massive connectivity, and highly reliable communication features, which are also crucial for healthcare IoT applications.

Aside from addressing the improvements to SimulatorOrchestrator outlined in the previous section, future work will address how influencing vehicular traffic patterns impacts healthcare outcomes, expanding the system’s capabilities to account for broader network effects on patient monitoring and response times. This integration would further validate the system’s applicability to real-time IoT-based healthcare systems, offering insights into emergency response dynamics and network management under various vehicular traffic conditions. Additionally, future work will also make use of real-world data from the Newcastle Urban Observatory (https://newcastle.urbanobservatory.ac.uk/, accessed on the 1 March 2025), replacing the generated patient data with data sampled from an urban scenario, albeit not strictly related to healthcare (pedestrian and vehicular mobility). At the time of writing, there were no publicly available datasets with real patient data streamed to a cloud data centre; for this work, we compromised using generated patient data, but incorporating real-world data will increase the utility of SimulatorOrchestrator.

In terms of security, existing IoT simulators, thus including the present one, often lack comprehensive communication and security features necessary for accurately modelling and mitigating real-time cybersecurity threats, such as battery-draining attacks. One key factor preventing many Osmotic simulators from adequately addressing security concerns is the lack of simulating the full OSI stack, particularly the lower OSI layers. For example, this work focussed on the Presentation and Session layers. As discussed within Section 2.2, in line with other Osmotic simulators, we did not address all OSI layers within SimulatorOrchestrator due to concerns with computational complexity, and chose the Presentation and Session layers, as they were the most relevant to this work. Addressing this gap, the IoTSimSecure framework, as discussed in [33], introduced a novel simulation tool specifically designed to mitigate battery depletion attacks and enhance the security and longevity of IoT devices in various smart environments, including healthcare. Future work will then outline which minor extensions are also required to capture the lower architectural layers required for the cybersecurity algorithms without needing the full OSI layer stack. As discussed above in Section 6, any simulator needs to address the trade-off between realism and scalability, as such adding further OSI layers is likely to negatively impact the real-world runtime of the simulator without providing any additional benefit in terms of the collected information. It will therefore be important to carefully determine if adding lower OSI layers will come at the cost of the scale of future experiments, or perhaps allowing the choice within SimulatorOrchestrator on which layers are used, and ’disabling’ the simulation of, for example, the Presentation layer if it is not needed for the cybersecurity analysis. Given the proposed approach’s potential, future work will address the possibility of extending the main architecture by designing a full-bridged orchestrator interconnecting different simulators together in a cohesive view: by considering STNs, these would require the simulator to support digital twins, not only receiving but also accepting traffic data.

## Figures and Tables

**Table 1:** Generated network configuration out of the IoT and RSU distributions within the Andrea Costa Scenario.

Network Component	Number
Edge	
Edge Networks	8
Total Active First Mile Hosts	16
MELs available per Host	3
Edge Switches	16
Core Switches	3
Backbone Network	
Edge Gateway Switches	8
SDWAN Router Switches	25
Cloud Gateway Switches	1
Cloud Network	
Core Switches	8
Aggregate Switches	556
Edge Switches	278
Cloud Hosts	278
MELs available per Host	1

**Table 2:** Patient digital twin parameters. We represent healthy ranges and starting conditions for the heart rate (HR), blood pressure (BP), and body core temperature (BCT) for the low-, medium-, and high-risk patients. The starting values shown are mean (and standard deviation σ).

	HR (bpm)	BP (mm Hg)	BCT (°C)
Healthy Ranges	50–110	110–220	36–38
Low Risk Starting Values	80 (σ 1.5)	112 (σ 2)	36.8 (σ 0.2)
Medium Risk Starting Values	70 (σ 20)	120 (σ 25)	37 (σ 0.5)
High Risk Starting Values	80 (σ 20)	90 (σ 20)	37 (σ 0.5)

**Table 3:** Packet rate from IoT to the cloud.

Configuration	Communications per Second
SPMB-Nearest	4.52
SPMB-Quietest	6.58
MCFR-Nearest	4.68
${\mathrm{MCFR-Quietest}}_{1}$	6.14
${\mathrm{MCFR-Quietest}}_{2}$	9.28

**Table 4:** The simulator real-world runtimes (RWRs) for each of the MEL routing policies, the time simulated (ST), and the difference between the two (DIF)—‘+’ indicates that the real-world time is longer than the simulated time, and conversely, ‘−’ indicates that the real-world time is shorter than the simulated time.

Configuration	ST (hh:mm:ss)	RWR (hh:mm:ss)	DIF (hh:mm:ss)
SPMB-Nearest	02:13:01	01:28:11	−00:44:50
SPMB-Quietest	01:29:48	00:42:46	−00:46:52
MCFR-Nearest	02:08:07	02:31:00	+00:22:53
${\mathrm{MCFR-Quietest}}_{1}$	01:35:53	00:52:46	−00:43:07
${\mathrm{MCFR-Quietest}}_{2}$	01:03:33	01:03:35	+00:00:02

**Table 5:** Correlating different simulation runtimes (RWRs) with different simulator configurations by varying the number of vehicles (vehs) or the number of patients (pats) and associated risk levels (Low = L; M = Medium; H = High). We pair these results to the total simulated time (ST) and its difference with RWR (DIF)—‘+’ indicates that the real-world time is greater than the simulated time, while ‘−’ indicates that the real-world time is less than the simulated time.

Configuration	ST (hh:mm:ss)	RWR (hh:mm:ss)	DIF (hh:mm:ss)	No. Comms
Part A: Varying IoT Device Number (vehs)				
118 vehs-60 pats	00:46:02	00:03:56	−00:42:06	11,915
236 vehs-60 pats	01:01:15	00:14:00	−00:47:15	20,614
354 vehs-60 pats	00:57:49	00:27:52	−00:29:57	27,344
472 vehs-60 pats	01:07:11	00:40:46	−00:26:25	32,472
590 vehs-60 pats	01:06:12	00:48:54	−00:17:18	34,553
709 vehs-60 pats	01:03:33	01:03:35	+00:00:02	35,314
Part B: Varying Communication Number				
20 L-risk Pats	01:01:09	00:25:29	−00:35:40	33,534
20 M-risk Pats	01:01:08	00:26:12	−00:34:56	33,594
20 H-risk Pats	00:59:24	00:35:54	−00:23:30	34,274
Part C: Removing the Patient Data				
709 vehs-0 pats	01:03:02	00:18:14	−00:44:48	32,954
Part D: Running the Simulator for 600 s with No Patients				
1664 vehs-0 pats	02:03:17	01:57:19	−00:05:58	67,022

## Data Availability

The codebase and datasets are available at https://github.com/LogDS/SimulatorOrchestrator/releases/tag/v1.0 (accessed on 1 March 2025). The results generated by the simulator can be found on OSF.io at https://osf.io/ykcjs/ (accessed on 1 March 2025).
